# Outcomes after readmission at the index or nonindex hospital following acute myocardial infarction complicated by cardiogenic shock

**DOI:** 10.1002/clc.23526

**Published:** 2021-01-07

**Authors:** Zhen Lin, Hedong Han, Yingyi Qin, Yuan Zhang, Daqing Yin, Cheng Wu, Xin Wei, Yang Cao, Jia He

**Affiliations:** ^1^ Department of Health Statistics Second Military Medical University Shanghai China; ^2^ Department of Respiratory and Critical Care Medicine, Jinling Hospital Nanjing University School of Medicine Nanjing China; ^3^ The Fifth Subcenter of Air Force Health Care Center for Special Services Hangzhou Wuxi China; ^4^ Department of Medical Management General Hospital of Central Theater Command Beijing China; ^5^ Department of Cardiology Virginia Commonwealth University Richmond Virginia USA; ^6^ Department of Clinical Epidemiology and Biostatistics School of Medical Sciences, Örebro University Örebro Sweden; ^7^ Department of Health Statistics Tongji University School of Medicine Shanghai China

**Keywords:** hospital costs, length of stay, myocardial infarction, patient readmission

## Abstract

Little is known about the prevalence and outcomes of readmission to nonindex hospitals after an admission for acute myocardial infarction complicated by cardiogenic shock (AMI‐CS). We aimed to determine the rate of nonindex readmissions following AMI‐CS and to evaluate its association with clinical factors, hospitalization cost, length of stay (LOS), and in‐hospital mortality rates.

**Hypothesis:**

Nonindex readmission may lead to worse in‐hospital outcomes.

**Methods:**

We reviewed the data of inpatients with AMI‐CS between 2010 and 2017 using the National Readmission Database. The survey analytical methods recommended by the Healthcare Cost and Utilization Project were used for national estimates. Multiple regression models were used to evaluate the predictors of nonindex readmission, and its association with hospitalization cost, LOS, and in‐hospital mortality rates.

**Results:**

Of 238 349 patients with AMI‐CS, 28028 (11.76%) had an unplanned readmission within 30 days. Of these patients, 7423 (26.48%) were readmitted to nonindex hospitals. Compared with index readmission, nonindex readmission was associated with higher hospitalization costs (p < .0001), longer LOS (p < .0001), and increased in‐hospital mortality rates (p = .0016). Patients who had a history of percutaneous coronary intervention, received intubation/mechanical ventilation, or left against medical advice during the initial admission had greater odds of a nonindex readmission.

**Conclusions:**

Over one‐fourth of readmissions following AMI‐CS were to nonindex hospitals. These admissions were associated with higher hospitalization costs, longer LOS, and higher in‐hospital mortality rates. Further studies are needed to evaluate whether a continuity of care plan in the acute hospital setting can improve outcomes after AMI‐CS.

## 1 INTRODUCTION

Acute myocardial infarction complicated by cardiogenic shock (AMI‐CS) is associated with respiratory failure, hemodynamic failure, and electrical instability, which may lead to hypoperfusion and organ failure.^1^ AMI‐CS was once a very deadly disease, with a survival rate of 40–44%,^2^, ^3^ however, in the past decade inpatient mortality has decreased significantly due to the use of reperfusion strategies and mechanical innovations in the treatment of AMI.^4^, ^5^ As survival rates have increased, improving post‐hospital outcomes is essential for patients with AMI‐CS. Rashmee et al.^6^ reported that patients with AMI‐CS have a high likelihood of adverse outcomes in the early stages after being discharged from the hospital.

With the escalating costs of healthcare, readmission has become an important health policy topic due to its relevance to the quality of medical services. Readmissions place a huge burden on medical resources and increase healthcare costs.^7^ Of the survivors of AMI‐CS who are discharged from the hospital, almost 20% are readmitted within 30 days.^8^ The Centers for Medicare & Medicaid Services (CMS) in the United States has set a quality measure to reduce the 30‐day readmission of patients with AMI.^9^ One unintended consequence of advances in treatment technology is that many patients are readmitted to hospitals other than the one where they initially received treatment, potentially fragmenting follow‐up care. Previous studies show that readmissions to a nonindex hospital by patients with acute stroke^10^ and by patients who underwent percutaneous coronary intervention (PCI)^11^ are associated with poorer outcomes. Similar results for patients undergoing different types of surgery have also been reported.^12^, ^13^, ^14^

Little is known about the prevalence and outcomes of readmission to a nonindex hospital following in patients diagnosed with AMI‐CS. Using the National Readmission Database (NRD) a nationally representative assessment of nonindex readmissions can be made. In this study, we determine the rate of readmission to nonindex hospitals following AMI‐CS and evaluate the association of clinical factors, hospitalization cost, length of stay (LOS), and in‐hospital mortality rates with nonindex readmission.

## 2 METHODS

### 2.1 Data source

The NRD is part of a set of databases and software tools developed for the Healthcare Cost and Utilization Project (HCUP). It is a unique and powerful database that supports various types of analyses on national readmission rates for all payers and for the uninsured. It estimates roughly 36 million discharges every year from more than 20 states in the United States.^15^ The NRD is publicly available, so this study was exempt from formal institutional review board approval, and informed consent is not required.

### 2.2 Study population

We used the International Classification of Diseases, Ninth Revision, Clinical Modification (ICD‐9‐CM) diagnosis codes or ICD‐10‐CM diagnosis codes to capture the data of patients with a primary admission for AMI (ICD‐9:410; ICD‐10:I21) who had an additional diagnosis of CS (ICD‐9:758.14, ICD‐10: R57.0). These codes have a specificity of 99.5% and a sensitivity of 72.4% for AMI,^16^ and a specificity of 99.3%, and a sensitivity of 60% for CS.^17^ We used the NRD variable “HOSP_NRD” to identify whether a patient was readmitted to the index hospital. The exclusion criteria included: (1) patients younger than 18 years, (2) patients who died during the index hospitalization, (3) patients who were discharged in December (as NRD does not provide cross‐year follow‐up data), and (4) elective readmissions.

### 2.3 Covariate assessment

We used the NRD variables to identify demographic characteristics such as age, sex, payer information, and income by postal code; and the hospital characteristics including the ownership of the hospital, the number of beds, and the location or teaching status. We used the Elixhauser Comorbidity Index (ECI) to account for the burden of 29 common comorbidities.^18^ Comorbidities, in‐hospital procedures, and in‐hospital complications were identified using ICD‐9‐CM and ICD‐10‐CM diagnostic or procedural codes (Table S1).^6^, ^7^, ^8^

### 2.4 Primary and secondary outcomes

Hospitalization cost, LOS, and in‐hospital mortality rate during unplanned readmissions within 30 days of the index admission were the primary study outcomes. The hospitalization cost was recalculated by cost‐to‐charge ratios, which was provided by HCUP. The secondary outcomes included the temporal trend in the prevalence of nonindex readmissions, in‐hospital outcomes during the 60 and 90 days readmission periods, predictors of readmission to a nonindex hospital, and the specific causes of readmission. The readmission causes were identified using Clinical Classification Software according to a previous study.^19^

### 2.5 Statistical analysis

We used the survey analytical methods recommended by the HCUP for national estimates.^15^ All readmissions were divided into readmissions at the discharging hospital (index readmissions) and readmissions to a different hospital (nonindex readmissions). The baseline characteristics during index admissions and the first readmissions were summarized based on the readmission hospital status. We used the chi‐square test to compare categorical variables and used the *t*‐test to compare the continuous variables between groups.

We categorized the reasons of 30 days readmission by cardiac cause and noncardiac cause. A multivariable logistic regression model was used to evaluate the predictors of readmissions to nonindex hospitals. Demographic characteristics, hospital characteristics, comorbidities, in‐hospital procedures, in‐hospital complications, LOS, and discharge disposition at the index hospital were incorporated into the model.

Different models were performed to elucidate the relationship between nonindex readmission and in‐hospital outcomes. In model A and model B, we included variables from the index admission. Demographic characteristics and hospital characteristics measured during the index admission were included in model A. Model B was adjusted for comorbidities, in‐hospital procedures, and in‐hospital complications measured during the index admission based on model A. In model C and model D, we included variables from the readmission. Demographic characteristics and hospital characteristics measured during the readmission were included in model C. Comorbidities and in‐hospital complications measured during the readmission were added to model C to derive model D.

Finally, we conducted several sensitivity analyses to confirm the primary outcomes. First, we repeated the analysis in patients divided into several causes of readmission, and in patients divided by history of PCI. Second, we used a propensity‐matched model to match patients readmitted to their index hospital and those readmitted to a nonindex hospital. The index and nonindex groups were matched using 1:1 matching protocol with a caliper of 0.1. Third, we evaluated the outcomes of 60 and 90 days readmission periods.

Two‐sided p values ≤ .05 were considered statistically significant. All statistical analyses were performed using SAS version 9.4 (SAS Institute Inc., Cary, NC).

## 3 RESULTS

### 3.1 Baseline characteristics

**FIGURE 1:**
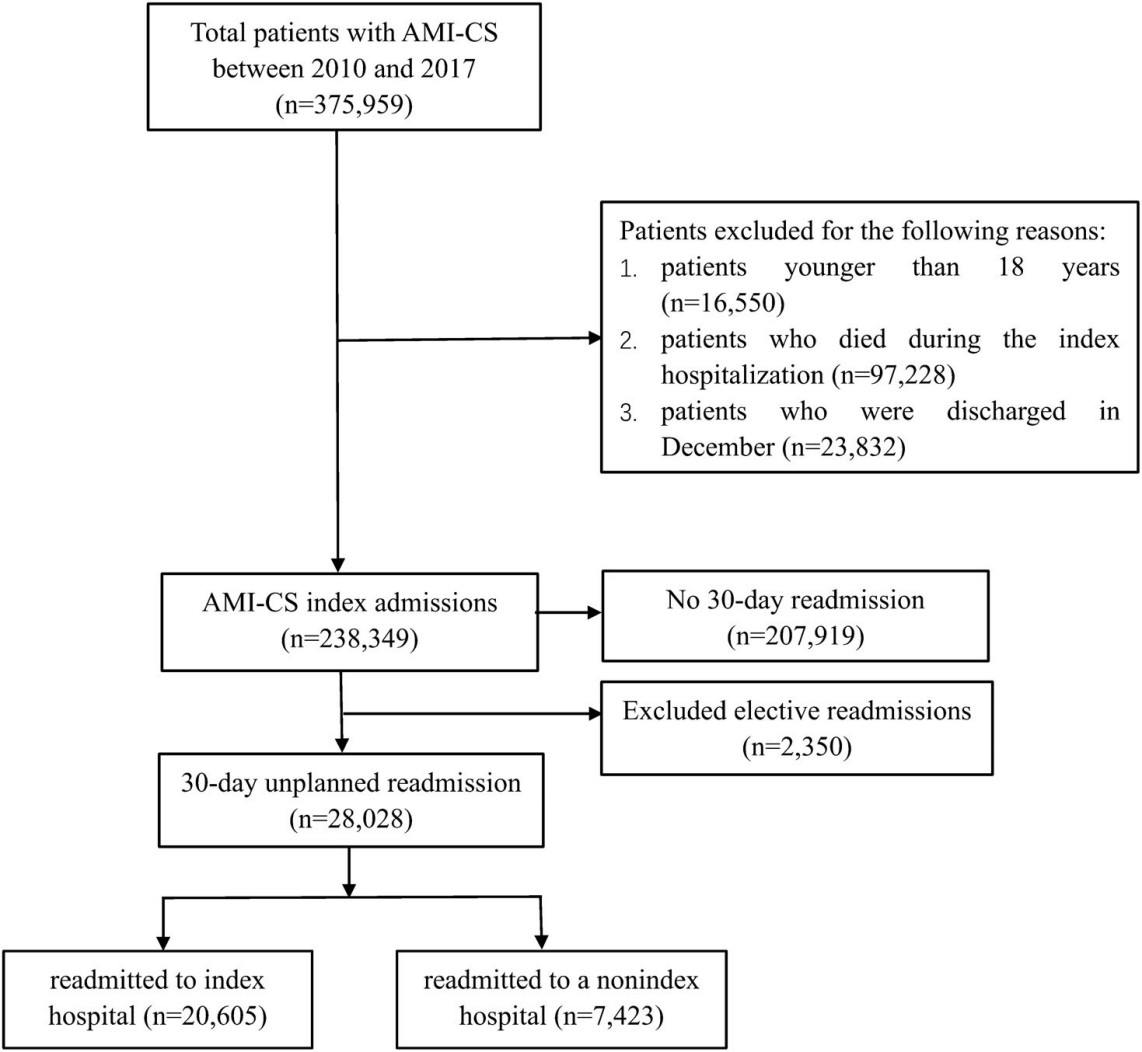
Selection flow diagram of target population

A weighted sample of 238 349 patients with AMI‐CS from 2010 to 2017 was identified, and the rate of unplanned 30 days readmissions was 11.76% (28 028 patients). Of these patients, 7423 (26.48%) were readmitted to a nonindex hospital (Figure 1). The prevalence of nonindex readmissions increased from 26.75% in 2010 to 27.17% in 2017 (P for trend = 0.9785) (Figure S1). Table 1 compares the patient characteristics recorded during index admissions between index and nonindex readmissions. Patients of nonindex readmissions were more likely to be older and residents of same state as the admitting hospital, and they had higher rates of a history of PCI, prior coronary artery bypass graft (CABG), and a history of stroke, as well as higher ECI scores. These patients were also less likely to receive PCI and CABG during the index hospitalization. When we compared the characteristics of 30‐day readmissions (Table S2), we found that patients of nonindex readmissions had higher ECI scores, and were more likely to go to private investor‐owned (proprietary) hospitals, smaller hospitals, urban nonteaching hospitals, or hospitals located at large metropolitan areas with at least 1 million residents. Patients of nonindex readmissions had a longer LOS (3.83 vs. 3.72 days, p = .0006), higher hospitalization costs ($10 224 vs. $9392, p < .0001), and higher in‐hospital mortality rates (9.39 vs. 7.30%, p = .0005).

**TABLE 1:** Comparison baseline characteristics during index admissions leading to index vs. nonindex readmission

	Index hospitals (N = 20 605)	Nonindex hospitals (N = 7423)	p‐value
Age, year, mean ± SE	67.47 ± 0.16	68.14 ± 0.24	.0203
Age, year (categories)			.0294
≤49	1701 (8.25)	520 (7.01)	
50–64	6614 (32.10)	2253 (30.36)	
65–79	8351 (40.53)	3142 (42.33)	
≥80	3940 (19.12)	1507 (20.31)	
Female sex, %	7996 (38.81)	2769 (37.30)	.1706
Weekend admission, %	5525 (26.82)	2022 (27.24)	.6631
Elective admission, %	735 (3.57)	334 (4.51)	.0268
Payer information, %			.0616
Medicare	13 085 (63.61)	4897 (66.03)	
Medicaid	2254 (10.96)	812 (10.95)	
Private insurance	3773 (18.34)	1227 (16.55)	
Self‐pay	784 (3.81)	293 (3.95)	
Other	676 (3.28)	187 (2.52)	
Resident of same state	19 373 (94.02)	7192 (96.90)	<.0001
Patient zip code income quartile			.0173
0–25th percentile	6349 (31.31)	2214 (30.30)	
26th–50th percentile	5389 (26.57)	1922 (26.31)	
51st–75th percentile	4921 (24.26)	1664 (22.77)	
76th–100th percentile	3621 (17.86)	1506 (20.62)	
Hospital characteristics			
Control or ownership of hospital			.0443
Government, nonfederal	2150 (10.44)	881 (11.88)	
Private, nonprofit	15 662 (76.01)	5467 (73.65)	
Private, invest‐own	2792 (13.55)	1074 (14.47)	
Hospital bed size			.0040
Small	1367 (6.63)	637 (8.58)	
Medium	4157 (20.17)	1607 (21.64)	
Large	15 081 (73.19)	5180 (69.78)	
Hospital urban–rural designation			<.0001
Large metro area > 1 million residents	11 244 (54.57)	4801 (64.68)	
Small metro area < 1 million residents	8402 (40.78)	2335 (31.46)	
Micropolitan area	876 (4.25)	246 (3.31)	
Not metropolitan or micropolitan	83 (0.40)	41 (0.55)	
Location/teaching status of hospital, %			.0056
Urban nonteaching	6694 (32.49)	2200 (29.64)	
Urban teaching	12 952 (62.86)	4935 (66.49)	
Rural	958 (4.65)	287 (3.86)	
Comorbidities			
Elixhauser comorbidity index	3.01 (1.65–4.53)	3.32 (1.98–4.82)	<.0001
Prior MI	2350 (11.40)	937 (12.63)	.0778
Prior PCI	1853 (9.00)	801 (10.79)	.0088
Prior CABG	1153 (5.60)	534 (7.20)	.0015
Prior stroke/TIA	821 (3.98)	379 (5.10)	.0080
Carotid artery disease	383 (1.86)	140 (1.89)	.9150
Smoking history	6626 (32.16)	2431 (32.75)	.5758
Dyslipidemia	9875 (47.92)	3620 (48.76)	.4356
Hypertension	12 351 (59.94)	4599 (61.96)	.0692
Diabetes	9202 (44.66)	3562 (47.99)	.0032
Drug abuse	696 (3.38)	237 (3.20)	.6799
Alcohol abuse	967 (4.69)	341 (4.60)	.8399
Depression	1753 (8.51)	675 (9.09)	.3295
Anxiety	1403 (6.81)	506 (6.81)	.9960
Congestive heart failure	1024 (4.97)	444 (5.99)	.0387
Metastatic cancer	227 (1.10)	70 (0.95)	.4249
In‐hospital procedures			
PCI	8502 (41.26)	2854 (38.45)	.0222
CABG	3568 (17.32)	1132 (15.25)	.0245
Intraaortic balloon pump	6860 (33.29)	2356 (31.74)	.1765
Long‐term VAD	130 (0.63)	7 (0.09)	<.0001
Short‐term VAD	472 (2.29)	221 (2.98)	.0903
Intubation/mechanical ventilation	5532 (26.85)	2379 (32.05)	<.0001
In‐hospital complications			
Acute renal failure	9520 (46.20)	3693 (49.75)	.0014
Pneumonia	3362 (16.32)	1280 (17.25)	.2403
Gastrointestinal bleeding	1036 (5.03)	388 (5.23)	.6792
Acute ischemic stroke/TIA	742 (3.60)	334 (4.50)	.0410
DVT/PE	19 121 (92.80)	6628 (89.30)	<.0001
Sepsis	2176 (10.56)	886 (11.93)	.0409
Atrial fibrillation	5490 (26.65)	2086 (28.10)	.1481
In‐hospital outcomes			
Index length of stay, day	10.20 (5.93–16.94)	10.39 (5.85–18.36)	.0491
Index cost, $,	45 272 (27944–73 385)	47 295 (27490–78 909)	.0041
Disposition			<.0001
Routine: home or self‐care	7731 (37.52)	2363 (31.84)	
Transfer to short‐term hospital	410 (1.99)	631 (8.50)	
Transfer to SNF, ICF, or other facility	6852 (33.25)	2759 (37.17)	
Home health care	5432 (26.36)	1555 (20.95)	
Against medical advice	177 (0.86)	113 (1.52)	

Abbreviations: APR‐DRG, all patient refined diagnosis related groups; DVT, deep venous thrombosis; ICF, intermediate care facility; MI, myocardial infarction; PCI, percutaneous coronary intervention; PE, pulmonary embolism; SE, standard error; SNF, skilled nursing facility; TIA, transient ischemic attacks; VAD, ventricular assist device.

### 3.2 Reasons for 30 day unplanned readmissions

Overall, the reasons of 30 day unplanned readmissions were similar between patients who rehospitalized to the index hospital and those who went to a different hospital. Table S3 shows the most common reasons for readmission to either index or nonindex hospitals. The most common noncardiac cause for readmission was infection in both patient groups, however, the rate was higher in patients who rehospitalized at a nonindex hospital (25.15 vs. 20.24%) The most common causes for cardiac readmission for both cohorts were heart failure, AMI, coronary artery disease (including angina), arrhythmia, and hyper/hypotension. These causes made up more than 90% of the cardiac readmissions.

### 3.3 Predictors of nonindex readmission

Multivariable analysis revealed that PCI (odds ratio [OR], 0.89; 95% confidence interval [CI], 0.79–0.99), CABG (OR, 0.82; 95% CI, 0.70–0.95), the presence of a long‐term ventricular assist device (OR, 0.11; 95% CI, 0.04–0.34), and deep venous thrombosis/pulmonary embolism (OR, 0.74; 95% CI, 0.63–0.88) were associated with a reduced likelihood of a nonindex readmission (Table 2). Patients initially admitted to hospitals that were private and nonprofit, had a high number of beds, or located in small metro areas with <1 million residents were less likely to be readmitted to a nonindex hospital. Compared to nonresidents, residents of the same state as the index hospital were more likely to be readmitted to a nonindex hospital. We found that patients initially admitted to urban teaching hospital were also more likely to be readmitted to a nonindex hospital. Patients who had a history of PCI (OR, 1.22; 95% CI, 1.05–1.42), received intubation or mechanical ventilation (OR, 1.27; 95% CI, 1.14–1.42), were transferred to a short‐term hospital (OR, 4.99; 95% CI, 3.96–6.29), or left against medical advice (OR, 1.99; 95% CI, 1.37–2.88) were more likely to be readmitted to a nonindex hospital.

**TABLE 2:** The predictors of nonindex readmission

	OR (95% CI)	p‐value
Age, per 1 year increase	1.00 (1.00, 1.01)	.1817
Female	0.93 (0.85, 1.03)	.1657
Weekend admission	1.02 (0.93, 1.13)	.6734
Elective admission	1.35 (1.08, 1.70)	.0084
Insurance		
Medicaid vs. medicare	1.01 (0.90, 1.15)	.8117
Private vs. medicare	0.89 (0.79, 1.02)	.0928
Self‐pay vs. medicare	1.02 (0.88, 1.18)	.8219
Others vs. medicare		
Resident of same state	2.07 (1.51, 2.84)	<.0001
Income quartile		
26st–50th vs. 1st–25th	1.05 (0.89, 1.25)	.5505
51st–75th vs. 1st–25th	0.98 (0.85, 1.13)	.7901
76st–100th vs. 1st–25th	1.22 (0.94, 1.59)	.1268
Hospital characteristics		
Control or ownership of hospital		
Private, nonprofit vs. Government, nonfederal	0.80 (0.68, 0.93)	.0044
Private, invest‐own vs. Government, nonfederal	0.89 (0.74, 1.07)	.2152
Hospital bed size		
Medium vs. small	0.81 (0.65, 1.00)	.0486
Large vs. small	0.76 (0.62, 0.92)	.0061
Hospital urban–rural designation		
Small metro area < 1 million residents vs. large metro area > 1 million residents	0.69 (0.62, 0.77)	<.0001
Micropolitan area vs. large metro area > 1 million residents	0.65 (0.49, 0.86)	.0032
Not metropolitan or micropolitan vs. large metro area > 1 million residents	1.07 (0.77, 1.48)	.6800
Location/teaching status of hospital		
Urban teaching vs. urban nonteaching	1.14 (1.02, 1.28)	.0268
Comorbidities		
Elixhauser comorbidity index		
1 vs. 0	1.04 (0.76, 1.42)	.8119
2 vs. 0	1.00 (0.74, 1.36)	.9942
≥3 vs. 0	1.22 (0.90, 1.66)	.2043
Prior MI	1.06 (0.93, 1.21)	.3696
Prior PCI	1.22 (1.05, 1.42)	.0114
Prior CABG	1.18 (0.99, 1.41)	.0587
Prior stroke/TIA	1.14 (0.93, 1.39)	.1993
Carotid artery disease	1.00 (0.72, 1.39)	.9851
Smoking history	1.08 (0.98, 1.21)	.1300
Dyslipidemia	0.98 (0.90, 1.07)	.6862
Hypertension	1.03 (0.93, 1.15)	.5363
Diabetes	1.04 (0.94, 1.15)	.4810
Drug abuse	0.85 (0.64, 1.15)	.2967
Alcohol abuse	0.87 (0.69, 1.10)	.2457
Depression	1.30 (0.95, 1.78)	.0984
Anxiety	0.77 (0.54, 1.10)	.1526
Congestive heart failure	0.97 (0.79, 1.20)	.7962
Metastatic cancer	0.81 (0.54, 1.23)	.3283
In‐hospital procedures		
PCI	0.89 (0.79, 0.99)	.0456
Coronary artery bypass grafting	0.82 (0.70, 0.95)	.0080
Intraaortic balloon pump	0.98 (0.87, 1.11)	.7581
Long‐term VAD	0.11 (0.04, 0.34)	.0001
Short‐term VAD	1.37 (0.97, 1.93)	.0698
Intubation/mechanical ventilation	1.27 (1.14, 1.42)	<.0001
In‐hospital complications		
Acute renal failure	1.01 (0.91, 1.11)	.8708
Pneumonia	0.97 (0.86, 1.09)	.6251
Gastrointestinal bleeding	0.96 (0.77, 1.18)	.6755
Acute ischemic stroke/TIA	1.09 (0.86, 1.39)	.4842
DVT/PE	0.74 (0.63, 0.88)	.0006
Sepsis	1.01 (0.87, 1.17)	.9028
Atrial fibrillation	1.03 (0.93, 1.14)	.5875
LOS	1.00 (1.00, 1.00)	.6286
Disposition		
Transfer to short‐term hospital vs. routine: home or self‐care	4.99 (3.96, 6.29)	<.0001
Transfer to SNF, ICF, or other facility vs. routine: home or self‐care	1.15 (1.02, 1.31)	.0253
Home health care vs. routine: home or self‐care	0.85 (0.75, 0.97)	.0161
Against medical advice vs. routine: home or self‐care	1.99 (1.37, 2.88)	.0003

Abbreviations: APR‐DRG, all patient refined diagnosis related groups; DVT, deep venous thrombosis; ICF, intermediate care facility; MI, myocardial infarction; PCI, percutaneous coronary intervention; PE, pulmonary embolism; SE, standard error; SNF, skilled nursing facility; TIA, transient ischemic attacks; VAD, ventricular assist device.

### 3.4 Impact of nonindex readmission on outcomes

After adjusting for demographic characteristics, hospital characteristics, comorbidities, in‐hospital procedures, and in‐hospital complications, model B revealed that patients with nonindex readmissions had $3422 higher hospitalization costs (95% CI, $2991–$3853, p < .0001), 0.49 days longer LOS (95% CI, 0.38–0.61 days, p < .0001), and higher in‐hospital mortality rates (OR = 1.29, 95% CI, 1.10–1.51, p = .0016). Similar results were found in other models (Table 3).

**TABLE 3:** Impact of nonindex readmission on outcomes

Readmission to a nonindex hospital vs. index hospital	Hospitalization costs, $			Length of stay, d			In‐hospital mortality		
	Difference	95% CI	p‐value	Difference	95% CI	p‐value	OR	95% CI	p‐value
Unadjusted model	3864	2566, 5162	<.0001	0.68	0.29, 1.08	.0006	1.32	1.13, 1.54	.0005
Model A	3768	3319, 4217	<.0001	0.63	0.51, 0.75	<.0001	1.33	1.14, 1.55	.0003
Model B	3422	2991, 3853	<.0001	0.49	0.38, 0.61	<.0001	1.29	1.10, 1.51	.0016
Model C	6209	5738, 6680	<.0001	1.23	1.11, 1.36	<.0001	1.45	1.23, 1.70	<.0001
Model D	4919	4465, 5373	<.0001	0.85	0.74, 0.97	<.0001	1.36	1.15, 1.6	.0003

*Note*: Model A: adjusting for demographic characteristics, hospital characteristics at the time of index admission. Model B: adjusting for demographic characteristics, hospital characteristics, comorbidities, in‐hospital procedures, and in‐hospital complications at the time of index admission. Model C: adjusting for demographic characteristics, hospital characteristics at the time of readmission. Model D: adjusting for demographic characteristics, hospital characteristics, comorbidities, and in‐hospital complications at the time of readmission.

Abbreviation: CI, confidence interval.

### 3.5 Sensitivity analyses

Table 4 shows the results of the sensitivity analysis. The results of the propensity‐matched model were similar to our primary outcomes, as were the results of outcomes during the 60 and 90 days readmission periods. When patients were grouped by cause of readmission, nonindex hospital readmissions were associated with higher costs and longer LOS for cardiac causes, but they were not associated with higher costs or longer LOS for noncardiac causes of readmission. In patients who underwent PCI, similar results as the primary analysis were observed.

**TABLE 4:** Sensitivity analyses for outcomes

Readmission to a nonindex hospital vs. index hospital	Hospitalization costs, $			Length of stay, d			In‐hospital mortality		
	Difference	95% CI	p‐value	Difference	95% CI	p‐value	OR	95% CI	p‐value
Propensity‐matched model	5419	4010, 6828	<.0001	0.99	0.55, 1.44	<.0001	1.37	1.12, 1.67	.0022
60 day readmission	2588	2320, 2856	<.0001	0.26	0.19, 0.34	<.0001	1.23	1.06, 1.42	.0074
90 day readmission	2100	1693, 2508	<.0001	0.20	0.08, 0.32	.0008	1.22	1.04, 1.42	.0141
Readmitted for cardiac reasons	7327	6713, 7940	<.0001	1.08	0.94, 1.22	<.0001	1.22	0.93, 1.59	.1495
Readmitted for noncardiac reasons	476	−650, 1601	.4076	0.05	−0.30, 0.40	.7671	1.34	1.10, 1.63	.0032
Readmitted for AMI	9184	6943, 11 424	<.0001	1.48	0.07, 2.89	.0387	1.26	0.82, 1.93	.2937
Readmitted for CAD	8190	6912, 9469	<.0001	1.87	1.48, 2.27	<.0001	9.19	2.74, 30.81	.0004
Patients who Underwent PCI									
Yes	3777	3246, 4309	<.0001	0.56	0.40, 0.72	<.0001	1.36	1.01, 1.82	.0419
No	3216	1071, 5361	.0033	0.16	−0.06, 0.98	.0823	1.25	1.04, 1.49	.0149

*Note*: All models were adjusting for demographic characteristics, hospital characteristics, comorbidities, in‐hospital procedures, and in‐hospital complications at the time of index admission.

Abbreviations: AMI, acute myocardial infarction; CAD, coronary artery disease; CI, confidence interval; PCI, percutaneous coronary intervention.

## 4 DISCUSSION

When CMS focus on 30 days readmission and reduce Medicare payments for these patients, hospitals reduce excess readmissions.^20^ However, hospital administrators can only identify readmissions to their own hospital, which may underestimate true readmission rates.^21^ Using the NRD, we can identify the index and nonindex readmission rates. Readmission within 30 days is common for patients who survive AMI‐CS.^6^, ^7^ In this study, approximately a fourth of those survivors were readmitted to a nonindex hospital. Our study demonstrates an association between rehospitalization to a nonindex hospital and the less favorable outcomes for patients with AMI‐CS. We found evidence of increased hospitalization costs, longer LOS, and increased in‐hospital mortality rates associated with readmission to nonindex hospitals.

As many patients with AMI‐CS survive their initial hospitalization, post‐discharge outcomes are important. More than half of survivors are readmitted or die within 1 year of their index admission, and patients with CS have a higher mortality rate than those without CS.^6^ In the early post‐discharge period, patients with CS have a greater risk of poor outcomes than patients without CS.^6^ Using the NRD, Shah et al. reported that 20.2% of AMI‐CS survivors in the US from 2013 to 2014 were readmitted within 30 days.^7^ Another study determined the rate of readmission to be 18.6%.^8^ While these studies address the rate and predictors of readmissions in patients with AMI‐CS, they do not report the relationship between readmissions and outcomes.

Previous studies have reported similar outcomes of readmission to a nonindex hospital for patients undergoing surgical procedures.^14^, ^22^ PCI is a common revascularization modality in patients with AMI‐CS,^23^ and more than half of patients in this study underwent PCI. We performed subgroup analysis based on whether patients had PCI or not and found that patients who underwent PCI had longer LOS and higher mortality rates. We found that patients readmitted to nonindex hospitals have a higher in‐hospital mortality rate, which differs from previous reports on the readmission outcomes of patients with other diseases such as colon cancer^24^ and cirrhosis.^25^

Evidence regarding predictors of readmission to a nonindex hospital in patients with AMI‐CS is lacking. In our study, requiring intubation or mechanical ventilation during the initial admission were associated with a higher likelihood of a nonindex readmission, which may be explained by the fact that these patients had a higher risk of having an acute emergency and presenting to the nearest hospital instead of a specialized hospital. For example, when patients have a relapse of AMI, they may go to the nearest hospital instead of to the index hospital. In our multivariable analysis, the number of beds in a hospital is a predicator for nonindex readmission. Patients who were initially admitted to a smaller hospital were more likely to be readmitted to medium or large hospitals. Therefore, small hospital readmission rates may be underestimated. In addition, patients who left against medical advice were more likely to be readmitted to a nonindex hospital. Patient compliance is an area of potential intervention. A survey by Herzig et al. showed that most readmissions are related to patient understanding and the patient's self‐management capabilities.^26^

In the multivariate analyses, the results were similar regardless of adjusting for the variables of index hospitalization (model B) or readmission (model D). The higher in‐hospital mortality rate during readmission to nonindex hospitals is not fully understood. Hua et al. believe that it may be due to incomplete knowledge of the patient, causing delays in diagnosis and treatment.^27^ Nonindex readmissions can also lead to repeated testing, which causes increased hospitalization costs. Studies have shown that continuity of care contributes to improved survival after readmission and reduced use of health care resources.^22^, ^28^ However, patients may receive acute treatment far from home, which can lead to fragmented and lower quality care. When patients are readmitted to a local hospital without a cardiovascular specialist, the available provider may not have experience managing the patients' complications.^10^

Fragmented care and repeated testing can lead to higher medical costs, but receiving treatment from a different doctor, especially one who specializes in cardiovascular disease, can lead to better outcomes for patients.^29^ Patients who receive treatment from more than physician benefit from fewer missed diagnoses and medical errors. Follow‐up care is also an important aspect of patient well‐being. Dickinson et al. reported that more than 70% of unplanned readmissions were related to complications and were preventable, which suggests that improvement of follow‐up care can reduce the risk of readmission and post‐discharge mortality.^30^ Furthermore, sharing electronic health records and having patient navigators may mitigate or offset some of the negative consequences of medical interruptions.^25^ A better understanding of readmission patterns is needed to help reduce unnecessary readmissions, and could be accomplished by the development of a national database of demographic, clinical, and administrative data from different hospitals.^31^

This study has several limitations. First, due to the use of administrative data, misclassifications or residual confounding may bias our results. Second, the NRD does not contain some important clinical information, such as medications or physiological data. Although we adjusted for several factors (patient characteristics and hospital characteristics) and performed some sensitivity analyses, some unknown bias is still possible. Third, only readmissions within the same state are calculated by the NRD. Thus, patients readmitted to hospitals in other states were not included in the dataset. In addition, we were unable to determine the impact or rate of inter‐hospital transfers of AMI‐CS patients readmitted to a nonindex hospital.

## 5 CONCLUSIONS

This study highlights the readmission burden after AMI‐CS. We found that readmission to a nonindex hospital occurs for more than a quarter of survivors of AMI‐CS, and is associated with higher hospitalization costs, longer LOS, and higher in‐hospital mortality rates. Decreasing readmission rates may result in reduced utilization of the health care system as well as improved outcomes for all patients with AMI. Further studies are needed to evaluate whether a continuity of care plan in the acute hospital setting may improve outcomes after AMI‐CS.

## CONFLICT OF INTEREST

All authors declare that they have no conflict of interest.

## AUTHOR CONTRIBUTIONS

Zhen Lin, Hedong Han, and Jia He designed the research. Zhen Lin, Hedong Han, and Yingyi Qin had full access to the data and conducted all analyses. Zhen Lin and Hedong Han wrote the article draft. Daqing Yin, Xin Wei, Cheng Wu, Yang Cao, and Jia He critically reviewed and revised the article. All authors contributed to the writing of the manuscript and read and approved the final manuscript. Jia He acted as the guarantor.

## Supporting information


**Appendix** S1: Supporting information

## Data Availability

Data was extracted from Nationwide Readmission Database . It is publicly available and can be accessed at hcup‐us.ahrq.gov.
