# Supplementary material for: Outcomes after readmission at the index or nonindex hospital following acute myocardial infarction complicated by cardiogenic shock
Source: Clin Cardiol. 2021 Jan 7;44(2):200–9. doi: 10.1002/clc.23526 (PMC7852161; doi:10.1002/clc.23526)
Supplement: Supplementary file 1 — Appendix S1: Supporting information [file CLC-44-200-s001.docx]

e-Figure 1. The prevalence of nonindex readmissions in AMI-CS.

**e-Table 1 acute myocardial infarction complicated by cardiogenic shock risk factors, severity indices and treatment variables**

| **Variables** | **ICD-9 codes** | **ICD-10 codes** |
| --- | --- | --- |
| Acute myocardial infarction | 410 | I21 |
| Cardiogenic shock | 785.51 | R57.0 |
| Prior MI | 412 | I25.2 |
| Prior PCI | V45.82 | Z98.61 |
| Prior CABG | V45.81 | Z95.1 |
| Prior stroke/TIA | V12.54, 438.x | Z86.73, I69.9 |
| Carotid artery disease | 433.10 | I65.29 |
| Smoking history | V15.82, 305.1 | F17.2, O99.33, Z87.891 |
| Dyslipidemia | 272.4 | E78.4, E78.5 |
| Anxiety | 311 | F32.9 |
| Gastrointestinal bleeding | 578.0, 578.1, 578.9 | K92.0,K92.1,K92.2 |
| PCI | Procedure code 00.66, 36.01, 36.06, 36.07,  and 36.09 | M6551-2, M6561-4, and M6571-2 |
| Coronary artery bypass grafting | Procedure code 36.1x | O1641-2,O6147,OA641-2, and OA647 |
| Intraaortic balloon pump | Procedure code 37.61 | 5A02110, 5A02210 |
| Long-term VAD | Procedure code 37.66, 37.52 | 02HA0QZ,02HA3QZ,02HA4QZ, 02RK0JZ, 02RL0JZ |
| Short-term VAD | Procedure code 37.68, 37.60, 37.62, or 37.65 | 02HA0RJ,02HA3RJ,02HA4RJ, 5A02116, 5A0211D, 5A0211D, 5A02216,5A0221D,02HA0RS, 02HA3RS, 02HA4RS, 5A02116, 5A02216, 02HA0RZ, 02HA3RZ, 02HA4RZ, 5A02116, 5A02216 |
| Ventilator use | Procedure code 9670，9671，9672，9605 | 5A1935Z, 5A1945Z, 5A1955Z, 5A09357, 5A09457, 5A09557 |
| Acute renal failure | 584.5 to 584.9 | N17 |
| Pneumonia | 486, 481, 482.8, 482.3 | J12-J18 |
| Acute ischemic stroke/TIA | 433.01, 433.11, 433.21, 433.31,  433.81, 433.91, 434.01,  434.11, 434.91, 435.x, 436 | I63, G45.9 |
| DVT/PE | 451.1, 451.2, 451.81, 451.9,  453.1, 453.2, 453.8,  453.9, 415.1 | I82, O22.3, O87.1, I88, Z86.711, Z86.718 |
| Sepsis | 038.0,038.1,0.38.2,0.38.3,038.4,0.38.8, 0.38.9, 995.92, 995.91, 003.1, 020.2, 022.3,036.2,036.3,054.5,098.89, 112.5, and 785.52 | A02.1,A20.0,A20.7,A21.7,A22.7,A24.1,A26.7,A28.2,A32.7,A39.14,A40,A41,A42.7,A48.3,B00.7,A54.8,B00.7,A54.8,B57.7,B57.6,B49,A49.9,P36,O75.3,O85,R65.0,R65.1,R57.2,R65.1 |

Abbreviation: SE, standard error; MI, myocardial infarction; PCI, percutaneous coronary intervention; CABG, coronary artery bypass grafting; TIA, transient ischemic attacks; VAD, ventricular assist device; DVT, deep venous thrombosis; PE, pulmonary embolism; SNF, skilled nursing facility; ICF, intermediate care facility.

**e-Table 2. Comparison baseline characteristics during readmissions leading to index vs nonindex readmission**

|  | Index Hospitals  (N=20605) | Nonindex Hospitals  (N= 7423) | P-value |
| --- | --- | --- | --- |
| Weekend admission, % | 5424 (26.32) | 1844 (24.84) | 0.1227 |
| Hospital Characteristics |  |  |  |
| Control or ownerlship of hospital |  |  | 0.0003 |
| Government, nonfederal | 2150 (10.44) | 955 (12.86) |  |
| Private, nonprofit | 15662 (76.01) | 5320 (71.67) |  |
| Private, invest-own | 2792 (13.55) | 1148 (15.47) |  |
| Hospital bed size, |  |  | <0.0001 |
| Small | 1367 (6.63) | 1203 (16.20) |  |
| Medium | 4157 (20.17) | 2249 (30.30) |  |
| Large | 15081 (73.19) | 3971 (53.50) |  |
| Hospital urban–rural designation |  |  | <0.0001 |
| Large metro area >1 million residents | 11244 (54.57) | 4630 (62.38) |  |
| Small metro area <1 million residents | 8402 (40.78) | 1853 (24.96) |  |
| Micropolitan area | 876 (4.25) | 730 (9.83) |  |
| Not metropolitan or micropolitan | 83 (0.40) | 210 (2.83) |  |
| Location/teaching status of hospital, % |  |  | <0.0001 |
| Urban nonteaching | 6694 (32.49) | 2952 (39.77) |  |
| Urban teaching | 12952 (62.86) | 3531 (47.57) |  |
| Rural | 958 (4.65) | 940 (12.66) |  |
| Comorbidities |  |  |  |
| Elixhauser comorbidity index | 2.87(1.56-4.39) | 3.03(1.72-4.59) | <0.0001 |
| Prior MI | 7161 (34.76) | 2744 (36.96) | 0.0297 |
| Prior PCI | 5744 (27.88) | 2082 (28.05) | 0.8802 |
| Prior CABG | 5427 (26.34) | 1939 (26.13) | 0.8381 |
| Prior stroke/TIA | 1011 (4.91) | 420 (5.66) | 0.1057 |
| Carotid artery disease | 255 (1.24) | 105 (1.41) | 0.5023 |
| Smoking history | 5773 (28.02) | 2034 (27.40) | 0.5567 |
| Dyslipidemia | 10261 (49.80) | 3578 (48.21) | 0.1587 |
| Hypertension | 13165 (63.89) | 4698 (63.29) | 0.6000 |
| Diabetes | 8995 (43.65) | 3496 (47.09) | 0.0012 |
| Drug abuse | 538 (2.61) | 178 (2.40) | 0.4972 |
| Alcohol abuse | 620 (3.01) | 231 (3.11) | 0.7842 |
| Depression | 1929 (9.36) | 696 (9.38) | 0.9757 |
| Anxiety | 1628 (7.90) | 602 (8.12) | 0.7369 |
| Congestive heart failure | 5944 (28.85) | 2122 (28.58) | 0.7970 |
| Metastatic cancer | 200 (0.97) | 49 (0.66) | 0.0598 |
| In-hospital complications |  |  |  |
| Acute renal failure | 5783 (28.07) | 2206 (29.72) | 0.0982 |
| Pneumonia | 2620 (12.71) | 1224 (16.49) | <0.0001 |
| Gastrointestinal bleeding | 863 (4.19) | 340 (4.58) | 0.3720 |
| Acute ischemic stroke/TIA | 550 (2.67) | 224 (3.02) | 0.3239 |
| DVT/PE | 20021 (97.16) | 7168 (96.57) | 0.1143 |
| Sepsis | 2120 (10.29) | 982 (13.23) | <0.0001 |
| Atrial fibrillation | 4781 (23.20) | 1640 (22.09) | 0.2160 |
| In-hospital outcomes |  |  |  |
| Readmit Length of stay, day | 3.72(1.83-7.35) | 3.83(1.78-7.92) | 0.0006 |
| readmit cost, $ | 9392(5156-19256) | 10224(5495-21670) | <0.0001 |
| readmit died | 1503 (7.30) | 696 (9.39) | 0.0005 |
| Disposition |  |  | <0.0001 |
| Routine: home or self-care | 8436 (40.96) | 2752 (37.12) |  |
| Transfer to Short-term Hospital | 320 (1.56) | 144 (1.94) |  |
| Transfer to SNF, ICF, or other facility | 5324 (25.85) | 2095 (28.25) |  |
| Home health care | 4867 (23.63) | 1628 (21.96) |  |
| Against medical advice | 123 (0.60) | 80 (1.08) |  |

Abbreviation: SE, standard error; SNF, skilled nursing facility; ICF, intermediate care facility.

**e-Table 3 reasons for 30-day unplanned readmissions**

| Cause of readmission | Index Hospitals (%) | Nonindex Hospitals (%) |
| --- | --- | --- |
| Cardiac | 44.56 | 43.57 |
| Noncardiac | 55.44 | 56.43 |
| Cause of noncardiac readmission | | |
| Infections | 20.24 | 25.15 |
| Respiratory | 10.31 | 10.19 |
| Gastrointestinal | 7.25 | 6.37 |
| Peripheral vascular disease | 6.08 | 4.62 |
| Non-specific chest pain | 5.47 | 4.96 |
| Renal disease | 4.76 | 4.70 |
| Bleeding | 5.62 | 4.92 |
| Transient ischemic attack/stroke | 3.37 | 3.50 |
| Genitourinary | 2.95 | 3.23 |
| Cause of cardiac readmission | | |
| Heart failure | 45.09 | 43.80 |
| Acute myocardial infarction | 14.27 | 18.24 |
| Coronary artery disease including angina | 11.35 | 9.97 |
| Arrhythmia | 11.92 | 9.63 |
| Hyper/hypotension | 9.81 | 11.57 |
| Pericarditis | 1.56 | 1.08 |
| Valve disorders | 1.29 | 1.46 |
| Conduction disorder | 0.50 | 0.75 |
